# XRCC5 VNTR, XRCC6 -61C>G, and XRCC7 6721G>T Gene Polymorphisms Associated with Male Infertility Risk: Evidences from Case-Control and In Silico Studies

**DOI:** 10.1155/2017/4795076

**Published:** 2017-03-21

**Authors:** Danial Jahantigh, Abasalt Hosseinzadeh Colagar

**Affiliations:** Department of Molecular and Cell Biology, Faculty of Basic Sciences, University of Mazandaran, Babolsar, Iran

## Abstract

We evaluate the association between genetic polymorphisms of *XRCC*5 VNTR, *XRCC*6 -61C>G, and *XRCC*7 6721G>T with male infertility susceptibility. A total of 392 men including 178 infertile males (102 idiopathic azoospermia and 76 severe oligozoospermia) and 214 healthy controls were recruited. *XRCC*6 -61C>G and *XRCC*7 6721G>T genotyping was performed by PCR-RFLP whereas *XRCC*5 VNTR was performed by PCR. The 2R allele and 2R allele carriers of *XRCC*5 VNTR polymorphism significantly decreased risk of male infertility. The mutant GG genotypes and carriers of the CG and GG genotypes of *XRCC*6 -61C>G showed increased risk for the male infertility. Furthermore, the G allele of the *XRCC*6 -61C>G was correlated with increased susceptibility to male infertility. Likewise, the T allele of the *XRCC*7 6721G>T polymorphism was associated with increased susceptibility to male infertility in azoospermia. In silico analysis predicted that the presence of tandem repeats in XRCC5 gene prompter can be sequence to bind to more nuclear factors. Also, rs2267437 (C>G) variant was located in a well-conserved region in *XRCC*6 promoter and this variation might lead to differential allelic expression. The *XRCC*7 6721G>T gene polymorphism occurred in an acceptor-splicing site, but this polymorphism has no severe modification on XRCC7 mRNA splicing. Our results indicate the association of *XRCC*5 VNTR, *XRCC*6 -61C>G, and *XRCC*7 6721G>T gene polymorphisms with male infertility in Iranian men.

## 1. Introduction

Male infertility is responsible for 40–50% infertility problems which affects up to one in six couples worldwide [1]. However, the exact etiology and pathogenesis of approximately half of the male infertility cases remains unknown yet, and it is well known as idiopathic infertility. Some evidences declared that DNA damage in human spermatozoa is associated with poor semen quality and low fertilization rates for both in vitro and in vivo fertility, suggesting that sperm DNA damage could be used as a potential predictor of fertility [2, 3].

Defects in DNA repair pathways during spermatogenesis have negative effects on the integrity of sperm DNA; could decrease quality and quantity of sperm including morphology, movement, and number; and cause infertility [4–6]. DNA damages, single strand breaks (SSBs), and double strand breaks (DSBs) are induced by exogenous and endogenous agents such as ionizing radiation (IR), oxidative and replication stress, and naturally programmed processes including meiotic and V(D)J recombination ([7–10]; Zha et al. 2007). The DSB is the most harmful form which should be repaired with homologuos recombination (HR) or predominantly nonhomologous end joining (NHEJ) [9, 10]. NHEJ involves binding of the Ku protein, Ku70 (XRCC6)/80 (XRCC5) heterodimer, to broken DNA ends, which induces inward translocation of Ku and recruits the DNA-PK catalytic subunit (DNA-PKcs) (XRCC7), to the ends of the DSB to form DNA-dependent protein kinase (DNA-PK) [10]. All components of the DNA-PK, including Ku70, Ku80, and DNA-PKcs, were found in the radiosensitive spermatogonia, so that the Ku70 and/or Ku80-deficient testis displays elevated levels of DSBs as well as enhanced apoptosis and reduced sperm production [11–15]. It is reported that Ku proteins are downregulated in early meiotic cells, but are again expressed in late pachytene and diplotene spermatocytes, and they mediate repair proficiency in somatic cells of the testis, thereby assuring the fidelity of spermatogenesis [11–13].

Strong evidences support the relationship between genetic polymorphism of genes involved in DNA repair pathways with extra sperm DNA damage and male infertility risk [16–21]. Among several genetic variants of the *XRCC*5, *XRCC*6, and *XRCC*7, a novel variable number of tandem repeats (VNTR) in the promoter region of *XRCC*5 and two single nucleotide polymorphisms (SNPs) *XRCC*6 -61C>G and *XRCC*7 6721G>T located in the promoter region of *XRCC*6 and intron 8 of *XRCC*7 genes, respectively, have been extensively studied in various disorders such as cancer [22–29] and autoimmune diseases [30, 31], but whether genetic variants in these repair pathway genes affect susceptibility to male infertility remains unknown.

Since the Ku protein, Ku70 (XRCC6)/80 (XRCC5), and DNA-PKcs (XRCC7) as critical components of NHEJ play important role in DNA integrity of spermatogenesis and can affect the offspring, we hypothesize that genetic variation of these genes may contribute to male infertility risk. Therefore, in current study, we evaluated possible relation between these common and functional polymorphisms with male infertility risk in a case-control study, and also, in silico analysis was carried out to investigate the effects of these SNPs on the interaction of several factors and motifs, involved in transcription and mRNA splicing.

## 2. Material and Methods

### 2.1. Patients and Control Samples

A total of 178 infertile patients, including 102 men with idiopathic nonobstructive azoospermia and 76 men with severe oligozoospermia (semen count less than 5 × 10^6^/mL), aged 24–39 years, and mean 31.6 ± 4.4 years, were recruited from the Fatemeh Zahra In Vitro Fertilization (IVF) Center in Babol, Iran, between 2013 and 2015 (Table 1). Semen samples were obtained by masturbation into sterile tubes after sexual abstinence for 2 to 3 days. Before semen analysis, a questionnaire was distributed to obtain information on smoking habits, alcohol use, use or abuse of other substances and drugs, and a history of reproductive system diseases. The samples with known causes of infertility were excluded; therefore, the patients with history of cryptorchidism, orchitis, obstruction of the vas deferens, varicocele, infectious diseases, drug abuse, diabetes mellitus, abnormal hormone profile (LH, FSH, and testosterone), Y-chromosome microdeletion, and abnormal karyotype were excluded from the study. So, only men with idiopathic infertility were included in the study. All patients underwent at least two semen analyses according to World Health Organization (WHO) guidelines after 3–5 days of sexual abstinence. The control group consisted of 214 men who were proven fertile with normozoospermia aged from 23 to 48 years. These studies were approved by the Ethics Committees of University of Mazandaran and informed consent was obtained from each subject before participation.

### 2.2. DNA Extraction, PCR, and RFLP

Genomic DNA was extracted from blood samples, which were collected in 2 ml EDTA_Na2_ tubes from patients and healthy controls, by using the commercial available kit (Roche, Germany). Extracted DNA was stored at −20°C. Fragments of the *XRCC5* 3R/2R/1R/0R-VNTR, *XRCC6* -61C>G promoter, and *XRCC7* 6721G>T amplified by polymerase chain reaction (PCR) using *XRCC*5-f/-r, *XRCC6*-f/-r, and *XRCC7*-f/-r primers, which were designed by [26], and PCR conditions, were described by [30].

DNA fragments of the *XRCC6* -61C>G and *XRCC7* 6721G>T were used in PCR-restriction fragment length polymorphism (PCR-RFLP) technique. Approximately 5 *μ*l (~0.1 *μ*g) of the *XRCC6* -61C>G and *XRCC7* 6721G>T amplified products were digested with 5 units of the *Ban*I (with cut site: 5′-GGCGCC) and *Pvu* II (with cut site: 5′-CAGCTG), respectively. Digestion reactions were performed at the total volume 10 *μ*l with incubation at 37°C for 16 h. PCR and/or digestion products were electrophoresed on a 3% agarose gel and visualized by ethidium bromide staining. All of the PCR and restriction reactants were purchased from CinnaGen Co, Iran. All of the electrophoresis materials were prepared from Merck Co, Germany.

### 2.3. In Silico Analysis

In silico analysis was performed to evaluate potential biological functions of two promoteric SNPs, rs6147172 and rs2267437, which are located in promoter region of *XRCC*5 and *XRCC*6 genes, respectively. Also, an intronic SNP, rs7003908, which is located in the intron 8 of *XRCC*7 gene, was subject of in silico analysis.

For detection of core promoter motifs, the DNA sequences that contain promoteric SNPs were screened by the prediction tools, EPD [32] and ElemeNT [33]. Also, SNPnexus [34] and PROMO [35] were used to find potential transcription factor binding sites in SNPs containing promoter sequences. The intronic sequence of *XRCC*7 gene that contains 6721G>T mutation was analyzed by Human Splicing Finder version 3, HSF3 [36], SpliceAid 2 [37], and SplicePort [38] to predict the effects of this variation on XRCC7 mRNA splicing. Moreover, the conservation of the DNA sequences containing *XRCC*6 -61C>G and *XRCC*7 6721G>T sites were also evaluated by Golden Path [39] and further illustrated by WebLogo [40].

### 2.4. Statistical Analysis

Hardy-Weinberg equilibrium (HWE) was calculated for both the infertile and control groups (http://www.oege.org/software/Hardy–Weinberg.html). All data were analyzed using SPSS software version 18. Differences in frequency of alleles and genotypes were analyzed using the chi-square test or Fisher's exact test. The odds ratio (OR) and 95% confidence intervals (CIs) were estimated. Two-tailed *p* < 0.05 was considered statistically significant. According to our results, sample power was computed using STATA 10 software.

## 3. Results

### 3.1. Demographic Analysis

Demographic and clinical characteristics of infertile patients and controls have been mentioned in Table 1. The infertile men were different from fertile men in sperm count, sperm motility, and sperm morphology. However, no differences were found between the two groups in terms of semen viscosity, smoking habit, and body mass index. About 57.3% of the infertile men were azoospermic and 42.6% were severe oligospermic.

### 3.2. VNTR Analysis

**Figure 1:**
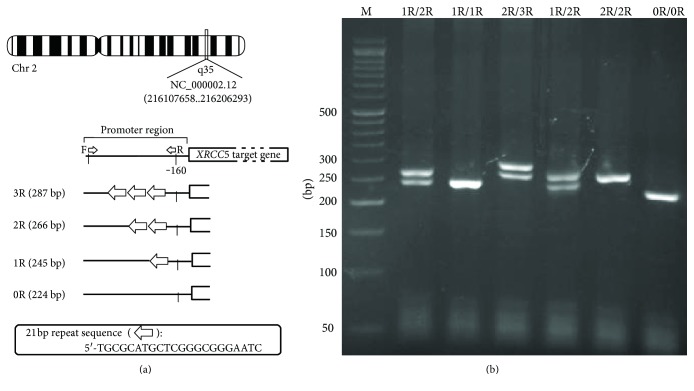
Schematic and PCR products of the *XRCC*5 VNTR: (a) schematic diagram of the XRCC5 gene showing the position of the VNTR polymorphism along with various alleles; (b) PCR products containing XRCC5 VNTR with various genotypes.

The *XRCC5* VNTR polymorphism analysis was performed for *XRCC5* promoter, which contains four different alleles with three 21 bp repeats (3R), two 21 bp repeats (2R), one 21 bp repeat (1R), and zero repeat (0R). Results of the PCR products by XRCC5-f/XRCC5-r primers in the agarose gel electrophoresis showed that size of fragments is 287 bp, 266 bp, 245 bp, and 224 bp for 3R, 2R, 1R, and 0R alleles, respectively (Figure 1).

The distributions of allelic and genotypic frequencies for *XRCC5* VNTR polymorphism have been summarized in Table 2. In VNTR polymorphism of *XRCC5* (rs6147172), among 10 probable genotypes, we observed 8 and 7 of them in control and male infertile groups, respectively. As it has been shown in Table 2, significant differences were observed between total infertile, azoospermic, severe oligozoospermic, and control groups in *XRCC5* VNTR genotypes and allele frequencies.

The 2R/2R genotype has the highest frequency in total infertile, azoospermic, severe oligozoospermic, and control groups (38.78, 28.65, and 26.45 versus 31.57 percent, resp.), in which differences between total and azoospermia patients compared to controls were statistically significant (*p* = 0.014, *p* = 0.011, resp.). Significant association between genotypic frequencies of 0R/2R and 1R/2R in total patients (*p* = 0.039, *p* = 0.014, resp.) and also azoospermia patients (*p* = 0.014, *p* = 0.015, resp.) with controls were observed. The frequency of individuals with 0R allele (0R/0R, 0R/1R, and 0R/2R genotypes) showed 1.6-fold increased risk of total infertility and was statistically different (*p* = 0.039). Moreover, high significant differences in frequencies of the 2R allele carriers (0R/2R+ 1R/2R+ 2R/2R+ 2R/3R), between total infertile and azoospermia, together with a slight association with severe oligozoospermia compared controls were found (*p* < 0.0001, *p* < 0.0001, and *p* = 0.013, resp.). The frequency of 2R allele of *XRCC5* VNTR polymorphism was found significantly higher than 0R allele in total, azoospermic, and severe oligospermic patients compared to control group (*p* = 0.0005, *p* = 0.001, and *p* = 0.01, resp.).

### 3.3. PCR-RFLP Analysis

**Figure 2:**
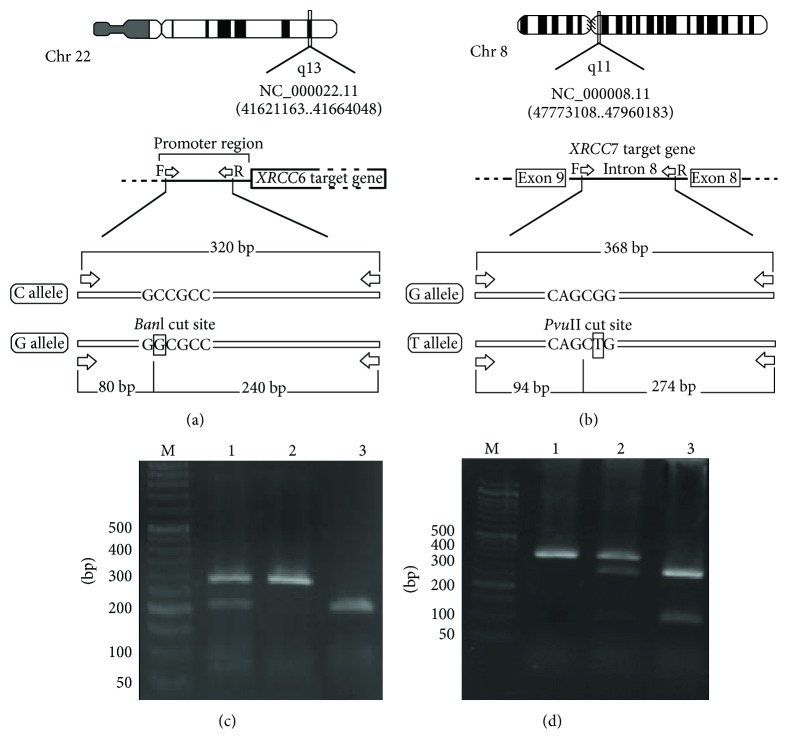
Schematic diagram and results of the PCR-RFLP products of XRCC6 and XRCC7: (a) schematic diagram of XRCC6 -61C>G, which showed *BanI* restriction enzyme site; (a′) polymorphism of PCR-RFLP products of the XRCC6 -61C>G gene in 1% agarose gel electrophoresis. Lane M: 50 bp DNA ladder; lane 1: CC homozygous genotype; lane 2: CG heterozygous genotype; lane 3: GG homozygous genotype; (b) schematic diagram of XRCC7 6721G>T which showed *PvuII* restriction enzyme site; (b′) polymorphism of PCR-RFLP products of the XRCC7 6721G>T gene in 1% agarose gel electrophoresis, lane M: 50 bp DNA ladder; lane 1: GG homozygous genotype; lane 2: GT heterozygous genotype; lane 3: TT homozygous genotype.

The 320 bp PCR product contains *XRCC6* -61C>G polymorphism digested by *Ban*I (Fermentas Co., Lithuania) to 240 and 80 bp fragments for G allele and a single 320 bp, no *Ban*I cleavage site, for C allele (Figures 2(a) and 2(a′)). The wild-type G allele of *XRCC7* 6721 G>T polymorphism was 368 bp size and had no *Pvu* II cleavage site, whereas mutant allele, T allele, was digested to 274 and 94 bp fragments (Figures 2(b) and 2(b′)).

The allele and genotype frequencies of *XRCC*6 and *XRCC*7 SNPs in male infertile patients and controls are summarized in Table 3. With regard to *XRCC*6 -61C>G, the CG genotype in azoospermia was approximately 2-fold higher than controls, which was significantly different (*p* = 0.003). The frequencies of the GG genotypes showed approximately 2.6, 3, and 2-fold increased risk of male infertility in total infertile, azoospermic, and severe oligospermic patients compared to control group, respectively, whose differences were statistically significant (*p* = 0.001, *p* = 0.001, and *p* = 0.043, resp.). Furthermore, the G allele frequencies in total infertile and azoospermia were approximately 1.5- and 2-fold higher than controls, and their differences were significant as well (*p* = 0.001 and *p* = 0.0002, resp.).

Finally, *XRCC*7 -6721G>T was evaluated in this study. Although mutant homozygotes and the T allele carriers (GT + TT) in azoospermic patients were 2.1 and 1.7-fold higher than control group, no significant differences were found (*p* = 0.118 and *p* = 0.247, resp.) (Table 2). Likewise, the frequency of allele T in azoospermia was significantly higher than controls, which was statistically different (*p* = 030).

### 3.4. Potential Biological Functions of XRCC5 VNTR, XRCC6 -61C>G, and XRCC7 6721G>T

Since the genetic variants in promoter region can affect gene transcription activity via altering the DNA-binding ability of transcription factors, we consequently evaluated the potential biological functions of *XRCC*5 VNTR (rs6147172) and *XRCC*6 -61C>G (rs2267437) using bioinformatics analysis.

**Figure 3:**
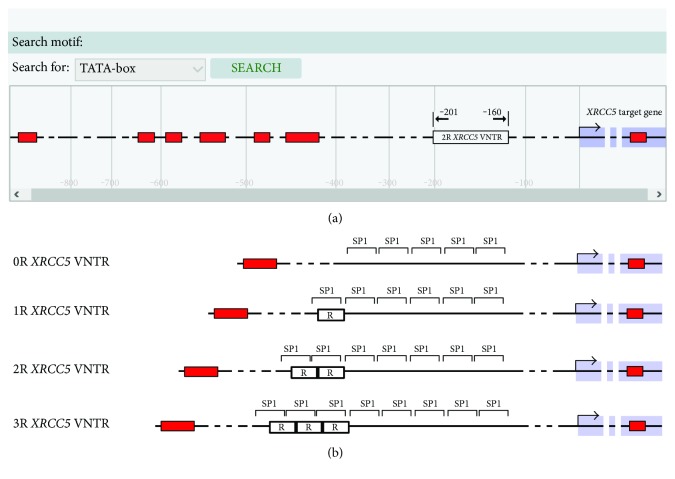
EPD report and schematic diagram of the different alleles of XRCC5 VNTR: (a) EPD report of the TATA-box situations and schematic diagram of VNTR (rs6147172) in the XRCC5 gene promoter; (b) schematic diagram of the different alleles of XRCC5 VNTR 0R, 1R, 2R, and 3R containing variable numbers of nuclear transcription factor Sp1.

The *XRCC*5 VNTR was scanned for transcriptional start site (TSS) and core promoter elements by EPD and ElemeNT. As shown in Figure 3(a), the VNTR of *XRCC*5 was located in downstream of the first TATA-box, −422 to −455, in the promoter region and TSS. The various tandem repeats of *XRCC*5 have not shown any change in number of core promoter elements, such as TATA-box, Inr, DPE, BRE, and DCE, but insertion of these tandem repeats moved these elements to upstream or downstream. The in vitro analysis demonstrated that four different alleles of *XRCC*5 VNTR 0R, 1R, 2R, and 3R contain variable numbers of nuclear transcription factor Sp1 (Figure 3(b)) [27].

**Figure 4:**
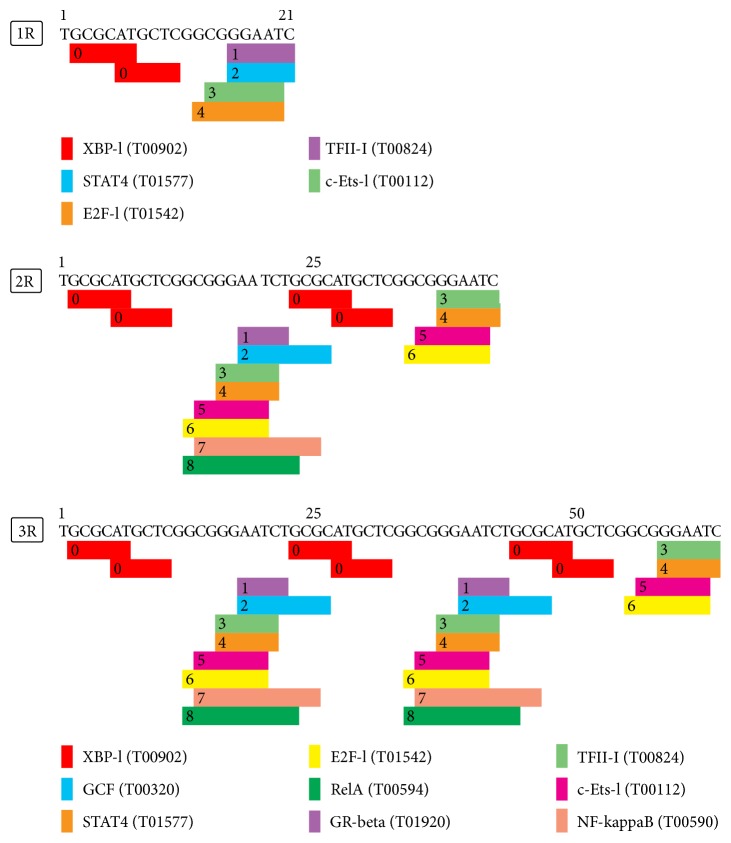
Potential binding sites for transcription factors of 1R, 2R, and 3R tandem repeat elements of the XRCC5 VNTR: XBP-1 = X-box binding protein 1; TFII-I = transcription factor II-I; STAT4 = signal transducer and activator of transcription 4; c-Ets-1 = c-E-twenty-six-1; E2F-1 = E2F transcription factor 1; GR-beta = glucocorticoid receptor-beta; GCF = GC factor; NF-kappaB = nuclear factor-kappaB; RelA = v-rel avian reticuloendotheliosis viral oncogene homolog A.

**Figure 5:**
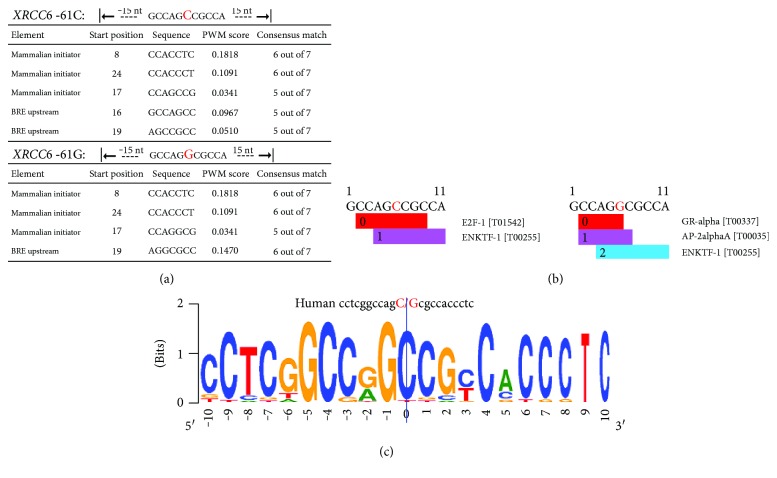
The prediction of core promoter elements, transcription factor binding sites, and sequence conservation of the XRCC6 -61C>G variant in the XRCC6 gene promoter: (a) prediction of core promoter elements via ElemeNT tool; (b) prediction of potential transcription factor binding sites; (c) illustration of sequence conservation of the DNA sequence around rs2267437 (C>G) locus.

Additional in silico analysis of these polymorphic tandem repeat elements revealed several potential binding sites for transcription factors such as TFII-I, E2F-1, STAT4, and NF-kappa B (Figure 4). Furthermore, the allele with more tandem repeats, 2R and 3R alleles, includes more binding site elements than the one-repeat allele, suggesting that the presence of more tandem repeats could increase the affinity of transcription factors to the region of the *XRCC*5 promoter (Figure 4). The prediction of core promoter elements via ElemeNT tool showed that rs2267437 (C>G) causes deletion of a BRE upstream element in the *XRCC*6 promoter, and also, this mutation increased the score of near BRE upstream (Figure 5(a)). The *XRCC*6 -61C>G variant was predicted by SNPnexus and PROMO tools to be located in the core recognition site of transcription factor E2F1 in *XRCC*6 gene promoter, and the transversion of allele C to allele G would lead to the loss of E2F1 binding site (Figure 5(b)). The rs189037 (C>G) was demonstrated by Golden Path tool to be located in a well-conserved region across multiple mammalian species and such conservation was further illustrated in WebLogo (Figure 5(c)).

**Figure 6:**
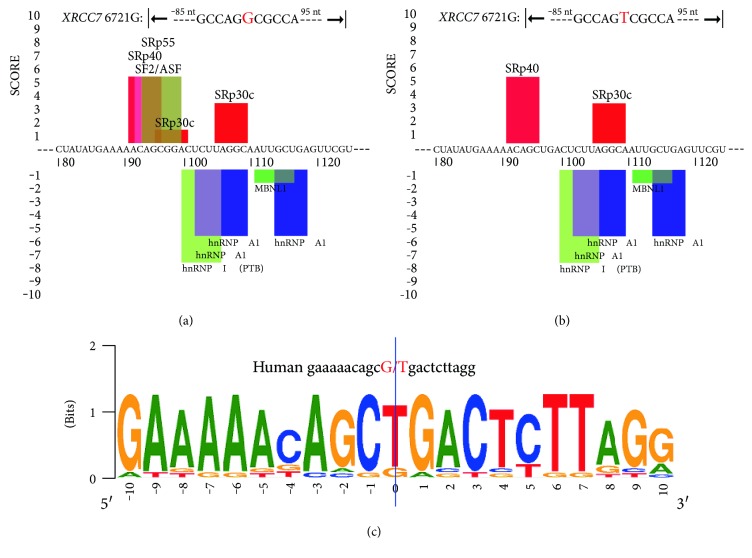
Enhancers and silencers of the XRCC7 6721G>T DNA sequence flanking motif and its sequence conservation: screening of the DNA sequence flankings of XRCC7 6721G>T for enhancer and silencer motif via SpliceAid 2 tool (a and a′); illustration of sequence conservation of the XRCC7 6721 across multiple mammalian species (b).

The prediction of the consequence of *XRCC*7 -6721G>T mutation via HSF 3 tool showed that this SNP occurred in an acceptor splicing site and the transversion of allele G to allele T causes the increase of HSF score of the mutation and also increased slightly the upstream acceptor splicing site's HSF score, but not created new cryptic splice acceptor site (Table 4(a)). The intronic variation of rs7003908 (G>T) increased branch point sequences as well (Table 4(b)). Screening of the DNA sequence flankings of rs7003908 (G>T) for enhancer motif, Exonic Splicing Enhancers (ESEs) and Intronic Splicing Enhancers (ISEs), and silencer motif, Exonic Splicing Silencers (ESSs) and Intronic Splicing Silencers (ISSs), by HSF 3 and SpliceAid 2 tools have shown almost same results. This in silico analysis revealed decrease, change, and removal of some of the ESEs such as SR (serine/argine-rich) proteins together with slight increase in ESE motifs from HSF for mutant allele T (Tables 4(c) and 4(d); Figures 6(a) and 6(a′)).

However, SpliceAid 2 has not shown any change in the silencer motifs (Figures 6(a) and 6(a′)), but HSF 3 displayed an ESS site broken for wild-type allele G and production of a new silencer motif site for mutant allele T which has potential alteration of splicing (Tables 4(e), 4(f), and 4(g)). Consistent with HSF 3 prediction, subsequent analysis with SplicePort tool showed that *XRCC*7 -6721G>T is presented in an acceptor site and it is not only decrease score of the mutation and near acceptor sites, but also able to influence on another acceptor or even donor sites (data not shown). The conservation of the *XRCC*7-6721 in across multiple mammalian species was assessed by GoldenPath tool and illustrated in WebLog (Figure 6(b)).

## 4. Discussion

To the best of our knowledge, the present study was the first demonstration that the *XRCC*5 VNTR, *XRCC6* -61C>G, and *XRCC7* 6721G>T polymorphisms are associated with susceptibility to male infertility. The results of our study showed that carriers of the 2R allele of *XRCC*5 VNTR were associated with a significantly decreased risk of male infertility. Moreover, the 2R allele of *XRCC*5 also significantly reduced the risk of male infertility (Table 2), suggesting that the presence of 2R allele in *XRCC*5 VNTR gene polymorphism may be a protective factor for male infertility. Besides, the mutant GG genotypes as well as carriers of the CG and GG genotypes showed increased risk for the male infertility. Furthermore, the G allele of the *XRCC*6 -61C>G variant was correlated with increased susceptibility to male infertility (Table 3). It suggests that the mutant allele G of *XRCC*6 -61C>G could be considered as a risk factor for male infertility susceptibility. Likewise, the T allele of the *XRCC*7 6721G>T polymorphism was associated with increased susceptibility to the male infertility in azoospermia (Table 3), which indicates the increased role of this polymorphism on male infertility risk.

Sperm DNA integrity is crucial for perfect transmission of genetic information; therefore, any sperm DNA damage may lead to male infertility despite the number, motility and morphology of spermatozoa, and, consequently, low fertilization rates, suggesting that it has a significant influence on the progeny [2–6]. Because of exogenous and endogenous agent's DNA breaks, a probable case in spermatogenesis together with malfunction of DNA repair mechanisms can affect normal sperm criteria and at last result in male infertility with azoospermia or oligospermia [4–6]. It is manifested that different polymorphic variants of genes encoding the proteins responsible for DNA repair were linked with the development of sperm DNA damage and male infertility [16–21].

NHEJ is a main mechanism for the removal of broad DNA double-strand breaks (DSBs) lesions and has a critical role in maintaining normal spermatogenesis and genetic stability [13, 15]. DNA-dependent protein kinase (DNA-PK) consists of a heterodimer DNA targeting subunit Ku70/Ku80, XRCC6/XRCC5, and catalytic subunit DNA-PKcs, XRCC7, which are imperative components of NHEJ which expresses in late spermatocytes of the testis, particularly [11, 13, 14]. All components of the DNA-PK were found in the radiosensitive spermatogonia, and also, it is demonstrated that Ku ensures the fidelity of spermatogenesis so that the Ku70 and/or Ku80-deficient testis displays elevated levels of DSBs as well as enhanced apoptosis and reduced sperm production [12, 14].

The *XRCC*5 VNTR polymorphism, rs6147172, was located in the promoter region and could affect the transcriptional activity of this gene [27, 41]. Polymorphism of rs6147172 has been implicated in susceptibility to several cancers including bladder cancer [27], acute myeloid leukemia (AML) [26], chronic myeloid leukemia (CML) [23], and autoimmune disease such as SLE [30]. In the current study, we observed that the frequency of 2R allele and 2R allele carriers had decreased risk of male infertility which can be considered as a genetic protective factor which was consistent with those previous roles in AML [26], CML [23], and SLE [30].

The strong evidence introduced VNTR sequences in promoter regions as regulatory elements which can bind to nuclear factors and affect transcriptional activity [42]. The promoter region of XRCC5 contains several copies of Sp1 recognition cis regulatory elements and its gene expression has Sp1-dependent manner (Figure 3(b)) [27, 43]. The VNTR polymorphism of XRCC5 can alter the number of Sp1 elements so that four different alleles of *XRCC*5 VNTR 3R, 2R, 1R, and 0R possess eight, seven, six, and five copies of Sp1 elements, respectively [41], which were able to modify the expression of XRCC5 [27, 43]. The in silico analysis of present study showed that the presence of tandem repeats in XRCC5 gene promoter can be sequenced to bind to more nuclear factors and probably affect transcriptional activity (Figure 4). This overactivity of XRCC5 leads to surplus DNA repair, which can increase the resistance of spermatogonia to genotoxic agents and interfere with DNA damage-dependent apoptosis and, thus, increase the likelihood for the development of normal sperm during spermatogenesis.

According to results of a newly meta-analysis which was performed on the XRCC6 SNP polymorphisms and cancer risk, the rs2267437 polymorphism was found to be associated with a significant increase in risks of overall cancers, breast cancer, RCC, and HCC, and it might increase the cancer risk in Asian population [25]. The result of current study showed a high association between genetic polymorphism of *XRCC*6 rs2267437 with male infertility, suggesting that this SNP might be a genetic risk factor for male infertility.

The *XRCC6* -61C>G polymorphism, rs2267437, was located in the promoter region and it is established that this SNP could influence the expression level and stability of the Ku70 protein in breast cancer cells and renal cell carcinoma tissues [44, 45]. Additional in silico analysis of current study to predict biological effects of rs2267437 (C>G) on XRCC6 expression showed that the *XRCC*6 -61C>G variant could cause deletion of a BRE upstream element in the XRCC6 promoter, firstly Figure 5(a), and the sequence region around rs2267437 (C>G) was predicated to be a DNA-binding site of E2F1, with the allele C to allele G change leading to the loss of this site, secondly Figure 5(b). Also, it was illustrated in WebLogo that rs2267437 (C>G) lies within a high conserved region across mammalian species, which indicates potential function for this variant (Figure 5(c)). Moreover, the sequence variation in the rs2267437 may affect binding activity of the adjacent CACCC box which is extended to their 4-5 upstream nucleotides and is known binding sites for SP1 and other Kruppel-like transcription factors [44, 46]. The binding and activity of SP1 and Kruppel-like transcription factors are heavily dependent on the SP1/Kruppel-like binding sites and adjacent sequences as well [47]. It has been demonstrated that E2F1 expression was required for the development of male germ cells [48, 49]. The sequence variation in the rs2267437 may abrogate the E2F1 binding site as well as affect binding activity of the adjacent CACCC box with transcription factors, resulting in decreased Ku70 expression level, and the DSB repair activity thus was affected, finally leading to spermatogenic failure.

Genetic polymorphisms of *XRCC7* 6721G>T were associated with an increased risk of glioma [22], bladder cancer [28], and SLE [30], while other studies reported that there were no significant associations between this polymorphism and risk of renal cell carcinoma and differentiated thyroid cancer [24, 29]. Our result further supports that the polymorphism of the *XRCC*7 6721G>T may be a genetic risk factor for azoospermic male infertile but not for total infertile and severe oligozoospermia (Table 3).

Defects and alterations in pre-mRNA splicing have been revealed as a common disease-causing mechanism in several studies [50, 51]. Because the *XRCC*7 6721G>T polymorphism, rs7003908, was located in the intron 8 of *XRCC*7 gene, auxiliary in silico analysis was performed to predict the effects of this variation on XRCC7 mRNA splicing. Bioinformatics analysis showed that although this SNP occurred in an acceptor splicing site and the transversion of allele G to allele T in *XRCC*7 6721 causes alterations such as increase in the score of the mutation and branch point sequences and can decrease, change, and remove some of the ESEs and also able to produce a new silencer motifs site which has potential alteration of splicing (Table 4 and Figure 6(a)), this variation cannot create new cryptic splice acceptor site, so this gene polymorphism has not severe modification on XRCC7 mRNA splicing. Moreover, it was illustrated in WebLogo that rs7003908 (G>T) was mapped within a slight conserved region across mammalian species, which indicates relative function for this variant (Figure 6(b)).

However, the functional significance of *XRCC*7 intron G6721T polymorphism is unknown; this single-nucleotide polymorphism might affect slightly the XRCC7 mRNA splicing and, thus, decrease the level of protein expression in splicing stages, which can affect DNA repair pathway and reduce the resistance of the cells against genotoxic agents in azoospermic male infertile patients' result. However, these possibilities should be investigated in future studies.

As an important pathway in the DNA damage repair network—NHEJ—XRCC5, XRCC6, and XRCC7 play a critical role in the maintenance of genetic integrity. Thus, it would be supposed that these three significant SNPs that affect sperm DNA integrity could also modify male infertility risk. However, our result showed significant association between *XRCC5*, *XRCC6*, and *XRCC7* gene polymorphisms and male infertility in an Iranian cohort. However, experimental studies are necessary to confirm such an assumption in the future.

In conclusion, this study provided evidences that the *XRCC5* VNTR, *XRCC6*, and *XRCC7* 6721G>T gene polymorphisms were associated with male infertility risk. Large cohort and diverse ethnicity studies as well as further functional analysis are needed to elucidate the biological mechanism of these polymorphisms of *XRCC5*, *XRCC6*, and *XRCC*7 in male infertility.

## Figures and Tables

**Table 1:** Demographic and clinical characteristics of participants.

Variable	Control (*n* = 214)	Patients			*p* value		
		Total (*n* = 178)	AS (*n* = 102)	Severe OS (*n* = 76)	Control versus total	Control versus AS	Control versus severe OS
*Age (years: mean ± SD)*	31.6 ± 4.4	32.3 ± 2.7	32.05 ± 4.1	31.8 ± 3.6	0.191	0.540	0.582

*Body mass index (kg/m^2^) (%)*							
≤24.9	27.1	25.8	23.5	28.9			
25–34.9	66.3	64.04	64.7	63.1			
≥35	6.5	10.1	11.7	7.8	0.201	0.119	0.689

*Smoking (%)*							
Yes	30.8	34.8	37.2	31.5			
No	69.1	65.1	62.7	68.4	0.401	0.257	0.904

*Semen analysis (%)*							
*(1) Sperm count ≤ 20 (million/mL)*	0	100	100	100	<**0.001**	<**0.001**	<**0.001**
*(2) Motility (grade a + b)*							
≥50	68.2	10.1	9.8	10.5			
<50	31.7	89.8	90.1	89.4	<**0.001**	<**0.001**	<**0.001**
*(3) Viscosity*							
≤30 min	57.9	73.03	72.5	73.6			
>30 min	42.05	26.9	27.4	26.3	0.316	0.251	0.694
*(4) Normal morphology*							
≥30%	34.5	13.4	11.7	15.78			
<30%	65.4	86.5	88.2	84.2	**0.001**	**0.004**	**0.004**

AS: azoospermia, OS: oligozoospermia.

**Table 2:** Genotype and allele frequencies of XRCC5 VNTR gene polymorphisms in controls and infertile patients.

Genotype	Control	Patients			*p* value [OR (95% CI )]			Study power %^a^		
	Total (*n* = 214) *n* (%)	Total (*n* = 178) *n* (%)	AS (*n* = 102) *n* (%)	Severe OS (*n* = 76) *n* (%)	Controls versus total patients	Controls versus AS	Controls versus severe OS	Controls versus total patients	Controls versus AS	Controls versus severe OS
0R/0R	4 (1.86)	11 (6.17)	7 (6.86)	4 (5.26)				49.03	48.45	23.85
0R/1R	12 (5.60)	21 (11.79)	13 (12.74)	8 (10.52)	0.510 [0.636 (0.165–2.445)]	0.519 [0.619 (0.144–2.659)]	0.360 [0.666 (0.128–3.469)]	51.85	50.14	24.19
1R/1R	17 (7.94)	33 (18.53)	22 (21.56)	11 (14.47)	0.531 [0.705 (0.195–2.552)]	0.668 [0.739 (0.185–2.945)]	0.589 [0.647 (0.133–3.141)]	**84.01**	**87.39**	31.32
0R/2R	23 (10.74)	16 (8.98)	6 (5.88)	10 (13.15)	**0.039** **[0.253 (0.068**–**0.937)]**	**0.014** **[0.149 (0.032**–**0.682)]**	0.299 [0.434 (0.090–2.094)]	5.109	19.25	5.054
1R/2R	64 (29.90)	39 (21.91)	22 (21.56)	17 (22.36)	**0.014** **[0.221 (0.066**–**0.744)]**	**0.015** **[0.196 (0.052**–**0.735)]**	0.080 [0.265 (0.060–1.173)]	38.46	28.53	18.35
2R/2R	83 (38.78)	51 (28.65)	27 (26.47)	24 (31.57)	**0.014** **[0.223 (0.067**–**0.739)]**	**0.011** **[0.185 (0.050**–**0.684)]**	0.095 [0.289 (0.067–1.243)]	51.44	50.39	15.53
0R/3R	—	—	—	—	—	—	—	—	—	—
1R/3R	3 (1.40)	—	—	—	0.570 [0.755 (0.286–1.991)]	0.130 [0.085 (0.003–2.072)]	0.238 [0.142 (0.005–3.642)]	10.33	−6.14	−3.894
2R/3R	8 (3.73)	7 (3.93)	5 (4.90)	2 (2.63)	0.142 [0.318 (0.068–1.468)]	0.224 [0.357 (0.067–1.879)]	0.191 [0.250 (0.031–1.998)]	3.348	3.566	7.091
3R/3R	**—**	—	—	—	—	—	—	—	—	—

0R carriers^∗^	39 (18.24)	48 (26.96)	26 (25.49)	22 (28.94)	**0.039** ^#^ **[1.656 (1.025**–**2.676)]**	0.136 [1.535 (0.872–2.699)]	0.050 [1.828 (0.998–3.348)]	49.40	27.39	43.89
0R noncarriers	175 (81.76)	130 (73.03)	76 (74.50)	54 (71.05)				49.50	27.41	43.94
1R carriers^∗∗^	96 (44.85)	93 (52.24)	57 (55.88)	36 (47.36)	0.145 [1.344 (0.902–2.004)]	0.067 [1.556 (0.968–2.502)]	0.495 [1.198 (0.714–2.009)]	27.14	40.07	3.265
1R noncarriers	118 (55.14)	85 (47.75)	45 (44.11)	40 (52.63)				27.14	40.07	3.265
2R carriers^∗∗∗^	178 (83.17)	113 (63.48)	60 (58.82)	53 (69.73)	**<0.0001** **[0.351 (0.219**–**0.562)]**	**<0.0001** **[0.288 (0.169**–**0.492)]**	**0.013** **[0.466 (0.2541**–**0.854)]**	**99.11**	**99.27**	63.23
2R noncarriers	36 (16.82)	65 (36.51)	42 (41.17)	23 (30.26)				**99.11**	**99.27**	63.25
3R carriers^∗∗∗∗^	11 (5.14)	7 (3.93)	5 (4.90)	2 (2.63)	0.570 [0.755 (0.286–1.991)]	0.928 [0.951 (0.321–2.813)]	0.372 [0.498 (0.108–2.303)]	3.19	3.087	3.761
3R noncarriers	203 (94.85)	171 (96.06)	97 (95.09)	74 (97.36)				3.18	3.085	3.762

0R allele	43 (10.04)	59 (16.57)	33 (16.17)	26 (17.10)				42.06	29.04	31.04
1R allele	113 (26.40)	126 (35.39)	79 (38.72)	47 (30.92)	0.384 [0.812 (0.509–1.297)]	0.733 [0.911 (0.532–1.558)]	0.217 [0.687 (0.379–1.245)]	44.12	55.1	8.872
2R allele	261 (60.98)	164 (46.06)	87 (42.64)	77 (50.65)	**0.0005** **[0.458 (0.295**–**0.710)]**	**0.001** **[0.434 (0.259**–**0.726)]**	**0.010** **[0.487 (0.281**–**0.845)]**	**81.51**	**83.92**	29.85
3R allele	11 (2.57)	7 (1.96)	5 (2.24)	2 (1.31)	0.142 [0.463 (0.166–1.293)]	0.372 [0.592 (0.187–1.871)]	0.136 [0.300 (0.061–1.464)]	4.507	4.188	2.69

^a^Power based on normal approximation with continuity correction.

^∗^0R carriers refer to individuals with the allele 0R including 0R/0R + 0R/1R + 0R/2R + 0R/3R, ^∗∗^1R carriers refer to individuals with allele 1R including 0R/1R + 1R/1R + 10R/2R + 1R/3R, ^∗∗∗^2R carriers refer to individuals with allele 2R including 0R/2R + 1R/2R + 2R/2R + 2R/3R, and ^∗∗∗∗^3R carriers refer to individuals with allele 3R including 0R/3R + 1R/3R + 2R/3R + 3R/3R.

^#^We calculated *p* value for the 0R carriers and compared the allele 0R carrier's individuals in the controls and patients against the entire individual without the allele 0R in the controls and patients, respectively; the other *p* value for the allele R carriers was calculated according to this formula.

**Table 3:** Genotype and allele frequencies of XRCC6 and XRCC7 gene polymorphisms in controls and infertile patients.

Genotype	Control	Patients			*p* value [OR (95% CI )]			Study power %^a^		
	Total (*n* = 214) *n* (%)	Total (*n* = 178) *n* (%)	AS (*n* = 102) *n* (%)	Severe OS (*n* = 76) *n* (%)	Controls versus total patients	Controls versus AS	Controls versus severe OS	Controls versus total patients	Controls versus AS	Controls versus severe OS
*XRCC6 -61C>G*										
CC	118 (55.14)	79 (44.38)	37 (36.27)	42 (55.26)				**52.31**	**86.09**	1.772
CG	75 (35.04)	62 (34.83)	44 (43.13)	18 (23.68)	0.348 [1.234 (0.794–1.918)]	**0.019** **[1.871 (1.107**–**3.160)]**	0.215 [0.674 (0.361–1.257)]	1.965	24.35	38.06
GG	21 (9.81)	37 (20.78)	21 (20.58)	16 (21.05)	**0.001** **[2.631 (1.434**–**4.827)]**	**0.001** **[3.189 (1.570**–**6.478)]**	**0.043** **[2.140 (1.021**–**4.484)]**	**82.26**	67.33	62.16
CG + GG	96 (44.85)	99 (55.61)	65 (63.72)	34 (44.73)	**0.034** **[1.540 (1.032**–**2.297)]**	**0.001** **[2.159 (1.329**–**3.508)]**	0.985 [0.995 (0.587–1.684)]	52.31	**86.09**	1.772
C	311 (72.66)	220 (61.79)	118 (57.84)	102 (67.10)				58.73	70.27	11.98
G	117 (27.33)	136 (38.20)	86 (42.15)	50 (32.89)	**0.001** **[1.643 (1.215**–**2.221)]**	**0.0002** **[1.937 (1.3653**–**2.748)]**	0.194 [1.303 (0.873–1.943)]	58.74	70.28	11.98
*XRCC7 6721G>T*										
GG	21 (9.81)	17 (9.55)	6 (5.88)	11 (36.84)				2.619	13.07	**99.72**
GT	104 (48.59)	74 (41.57)	41 (40.19)	33 (34.21)	0.720 [0.879 (0.434–1.779)]	0.518 [1.379 (0.519–3.664)]	0.235 [0.605 (0.264–1.386)]	24.88	24.45	52.76
TT	89 (41.58)	87 (48.87)	55 (53.92)	32 (28.94)	0.599 [1.207 (0.597–2.442)]	0.118 [2.162 (0.821–5.691)]	0.376 [0.686 (0.298–1.580)]	26.71	49	43.56
GT + TT	193 (90.18)	161 (90.44)	96 (94.11)	65 (63.15)	0.930 [1.030 (0.525–2.019)]	0.247 [1.740 (0.680–4.455)]	0.268 [0.643 (0.294–1.405)]	2.618	13.07	**99.72**
G	146 (34.11)	108 (30.33)	53 (25.98)	55 (36.18)				9.847	7.13	2.466
T	282 (65.88)	248 (69.66)	151 (74.01)	97 (63.81)	0.207 [1.213 (0.8981–1.639)]	**0.030** **[1.505 (1.039**–**2.180)]**	0.719 [0.931 (0.633–1.370)]	9.847	7.129	2.466

^a^Power based on normal approximation with continuity correction.

**Table:** (a) HSF matrices

Sequence position	cDNA position	Splice site type	Motif	New splice site	Wild type	Mutant	If cryptic site use, exon length variation	Variation (%)
83	+83	Acceptor	TATGAAAAACAGCG	TATGAAAAACAGCT	75.52	76.12	NA	+0.79
94	+94	Acceptor	GCGGACTCTTAGGC	GCTGACTCTTAGGC	75.48	78.56	NA	+4.08

**Table:** (b) Branch points

Sequence position	cDNA position	Branch point motif	CV for reference sequence (0–100)	CV for mutant sequence (0–100)	Variation
93	+93	AGCGGAC	71.15	86.24	+15.9

**Table:** (c) ESE Finder matrices for SRp40, SC35, SF2/ASF, and SRp55 proteins

Sequence position	cDNA position	Linked SR protein	Reference motif (value 0–100)	Linked SR protein	Mutant motif (value 0–100)	Variation
92	+92	SF2/ASF (IgM-BRCA1)	CAGCGGA (90.08)	SF2/ASF (IgM-BRCA1)	CAGCTGA (77.77)	−13.66%
92	+92	SF2/ASF (IgM-BRCA1)	CAGCGGA (90.08)	SF2/ASF	CAGCTGA (78.10)	−13.29%
92	+92	SF2/ASF	CAGCGGA (89.23)	SF2/ASF (IgM-BRCA1)	CAGCTGA (77.77)	−12.84%
92	+92	SF2/ASF	CAGCGGA (89.23)	SF2/ASF	CAGCTGA (78.10)	−12.47%
93	+93	SRp55	AGCGGA (76.48)			Site broken −100
95	+95	SF2/ASF (IgM-BRCA1)	CGGACTC (74.23)			Site broken −100
96	+96	SC35	GGACTCTT (75.23)			Site broken −100

**Table:** (d) ESE motifs from HSF

Sequence position	cDNA position	Linked ESE protein	Reference motif (value 0–100)	Linked ESE protein	Mutant motif (value 0–100)	Variation
94	+94	9G8	GCGGAC (77.99)	9G8	GCTGAC (78.59)	+0.77%

**Table:** (e) Silencer motifs

Sequence position	cDNA position	Sironi motif reference	Reference silencer (value 0–100)	Sironi mutant motif	Mutant silencer (value 0–100)	Variation
89	+89			Motif 1: CTAGAGGT	AAACAGCT (66.19)	New site
91	+91	Motif 1: CTAGAGGT	ACAGCGGA (65.75)			Site broken 0.66

**Table:** (f) hnRNP motifs

Sequence position	cDNA position	Linked hnRNP protein	Reference motif (value 0–100)	Linked hnRNP protein	Mutant motif (value 0–100)	Variation
92	+92	hnRNP A1	CAGCGG (66.67)			Site broken –100

**Table:** (g) Interpreted data

Predicted signal	Prediction algorithm	cDNA position	Interpretation
ESS site broken	(1) Sironi et al., Motif 1	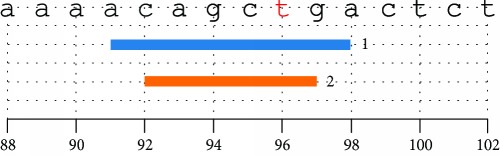	Alteration of an intronic ESS site Potential alteration of splicing
	(2) HSF matrices-hnRNP A1		
